# Urinary Protein and Peptide Markers in Chronic Kidney Disease

**DOI:** 10.3390/ijms222212123

**Published:** 2021-11-09

**Authors:** Natalia Chebotareva, Anatoliy Vinogradov, Valerie McDonnell, Natalia V. Zakharova, Maria I. Indeykina, Sergey Moiseev, Evgeny N. Nikolaev, Alexey S. Kononikhin

**Affiliations:** 1 Nephrology Department, Tareev Clinic, Sechenov First Moscow State Medical University, Trubezkaya, 8, 119048 Moscow, Russia; valerie_mcdonnell@yahoo.com (V.M.); avt420034@gmail.com (S.M.); 2 Department of Internal Medicine, Lomonosov Moscow State University, GSP-1, Leninskie Gory, 119991 Moscow, Russia; anatoliy_vinogradov@list.ru; 3 Emanuel Institute for Biochemical Physics, Russian Academy of Science, Kosygina Str., 4, 119334 Moscow, Russia; nvzakharova@yandex.ru (N.V.Z.); mariind@yandex.ru (M.I.I.); 4 Skolkovo Institute of Science and Technology, Bolshoy Boulevard 30, Bld. 1, 121205 Moscow, Russia

**Keywords:** biomarkers, urine, proteomics, chronic kidney disease

## Abstract

Chronic kidney disease (CKD) is a non-specific type of kidney disease that causes a gradual decline in kidney function (from months to years). CKD is a significant risk factor for death, cardiovascular disease, and end-stage renal disease. CKDs of different origins may have the same clinical and laboratory manifestations but different progression rates, which requires early diagnosis to determine. This review focuses on protein/peptide biomarkers of the leading causes of CKD: diabetic nephropathy, IgA nephropathy, lupus nephritis, focal segmental glomerulosclerosis, and membranous nephropathy. Mass spectrometry (MS) approaches provided the most information about urinary peptide and protein contents in different nephropathies. New analytical approaches allow urinary proteomic–peptide profiles to be used as early non-invasive diagnostic tools for specific morphological forms of kidney disease and may become a safe alternative to renal biopsy. MS studies of the key pathogenetic mechanisms of renal disease progression may also contribute to developing new approaches for targeted therapy.

## 1. Introduction

According to The Kidney Disease: Improving Global Outcomes (KDIGO) criteria, chronic kidney disease (CKD) is defined as an abnormality in kidney structure or function present for more than 3 months, with health implications [1,2]. CKD is an independent risk factor for death, cardiovascular disease, end-stage renal disease, and acute kidney injury [3,4,5,6,7] and has a global prevalence of 11–13% [8]. CKD is a socially significant problem due to the high risk of early disability from the disease and the need for high-cost treatments in the case of end-stage renal failure, such as hemodialysis, peritoneal dialysis, and kidney transplants [9,10]. The three most common causes of CKD are diabetes mellitus, hypertension, and glomerulonephritis, especially with nephrotic syndrome [11]. Kidney diseases can have similar clinical symptoms and may range from mild and benign to progressive with rapid end-stage renal disease development. The severity of the clinical manifestations, however, does not always correspond to the severity of renal damage, which can be determined by renal biopsy [12]. The majority of patients undergo a single kidney biopsy to determine the morphological form of kidney disease. In sporadic cases, the biopsy is repeated to assess the effectiveness of therapy and prognosis. However, the assessment of the regression of nephropathic activity is crucial for the dynamic assessment of treatment, including the treatment’s effectiveness, optimization, and prognosis. 

Urine proteomic analysis is a much safer option compared to a biopsy and has good potential for developing non-invasive diagnostic methods. Urine analysis has several advantages compared to blood proteomic analysis [13]. Firstly, the urine proteome is not very complicated and mainly contains proteins and peptides of renal origin (up to 70%). On the contrary, kidney damage markers comprise only a small fraction of the highly diverse plasma/serum proteome, making their analysis in the latter challenging. Secondly, it is much easier to normalize the concentration of a protein biomarker in the urine than in the blood—for example, based on the concentration of creatinine [14]. Thirdly, urine collection is simple and non-invasive. Finally, urine samples are stable at a temperature of −20 °C and are suitable for proteomic analysis even after years of storage [15]. The aforementioned advantages of urine make it a popular subject for the search for protein markers for various pathologies [16]. These pathologies include renal and genitourinary pathologies and pathologies associated with proteinuria, such as kidney diseases [17,18,19]; bladder, prostate, and ovarian cancers [20,21,22,23]; diabetic nephropathy [24]; and pre-eclampsia [25,26,27]. Urinary protein markers have also been described for colon and lung cancers [28,29], cholangiocarcinoma [30], cardiovascular diseases [31], autoimmune diseases [32], and infectious diseases [33]. Nevertheless, the urine proteome should be most informative for renal pathologies and may present a fingerprint of different kidney diseases [34,35,36,37,38,39] (Table 1). 

However, despite a large number of studies, there are still no reliable kidney-disease-specific biomarkers that can be accurately reproduced in different studies. The various factors affecting proteome composition include the collection conditions and regime (morning, daily, variability over several days, etc.), physical activity, nutrition, the anatomical features of the urinary tract (the absence of one kidney, etc.), sex, and age [40,41,42,43]. All of these factors should be taken into account when comparing the results of different studies. In general, combining the markers of specific nephropathies outlined in various studies could facilitate better progress in the creation of highly specific differentiating panels for possible clinical use after multi-stage prospective validation [44]. 

Mass spectrometry (MS)-based approaches, which feature a high multiplexing capacity, are the most unbiased and sensitive instruments and have already provided most of the currently known information about urine peptide and protein contents in different nephropathies, as well as potential biomarker panels for various diseases [37,38,39]. A number of MS methods have been successfully applied (Table 1). The most commonly used approaches include matrix-assisted laser desorption/ionization time-of-flight (MALDI-TOF), capillary electrophoresis (CE), and liquid chromatography (LC) MS. The most advanced approaches with isobaric or tandem mass tags for relative and absolute quantitation (iTRAQ and TMT) facilitate the identification of markers among commonly present proteins and peptides when their amounts vary significantly. In general, the listed untargeted MS approaches are the most appropriate for the primary search of potential biomarkers, whereas targeted MS and immunoassays can be used for further validation.

**Figure 1:**
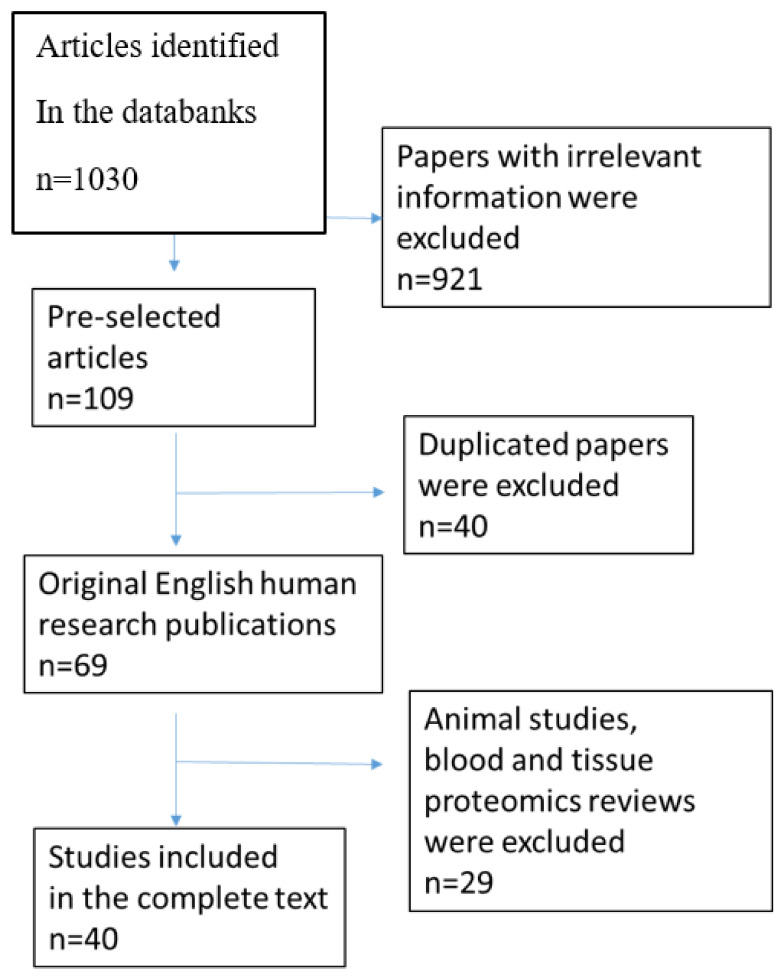
Study flow chart.

This review summarizes data from numerous studies of the urine proteome in nephropathies associated with CKD, with a focus on recent studies from 2015 to 2021. The electronic databases MEDLINE, PubMed, and Cochrane were searched using keywords such as “proteomics”, “peptidomics”, “biomarkers”, “chronic kidney disease”, “urine”, “membranous nephropathy” “IgA nephropathy”, “focal segmental glomerulosclerosis” “minimal-change disease”, “diabetic nephropathy”, and “lupus nephritis”. The reference lists of articles were also investigated to explore related literature. The bibliographic information of 1030 retrieved articles was analyzed, and papers with irrelevant or unreliable information, those unavailable in full text, and those not in English were deleted. After deleting all duplicate references, 69 articles remained. A flow chart is outlined in Figure 1.

**Table 1:** Urine proteome studies in different types of nephropathies.

Nephropathy Types	Method	Number of Patients	The Main Biomarkers	Functions of Proteins/Main Processes	References
Chronic kidney disease	CE-MS	4766	CKD 273 classifier ↓ fragments of different collagens, ↑ A1AT, serum albumin, hemoglobin α chain, fibrinogen α chain, uromodulin, Na^+^/K^+^-ATPase γ chain, and membrane-associated progesterone receptor component 1	CKD progression and fibrosis accumulation	Good et al., 2010 [45]
	CE-ESI-TOF MS	1028	CKD 273 classifier validation		Puntillo et al., 2017 [46]
	CE-ESI-TOF MS	1990	CKD 273 classifier validation		Schanstra et al., 2015 [18]
	CE-MS	435	FPP_29BH classifier ↑ cathepsin D, MMP-2, collagenase 3, MMP-14, α-2-HS-glycoprotein, fetuin-A, and 19 different collagen peptide fragments	Fibrosis accumulation	Catanese et al., 2021 [47]
FSGS/MCD	2D-DIGE-MS	49	A1AT, transferrin histatin-3 39S ribosomal protein L17 ↓ (FSGS vs. MCD) calretinin ↑ (FSGS vs. MCD)	Modulating immunity, inflammation and apoptosis; abnormal permeability of GBM, cell proliferation and differentiation	Perez et al., 2017 [48]
	2D-LC-MS/MS	30 FSGS 30- MCD	↑ Ubiquitin-60S ribosomal protein L40 (UBA52) (FSGS vs. MCD)	Protein degradation, stress response, and overexpression of UBA52 ameliorated the cell-cycle arrest	Wang et al., 2017 [49]
	LC-MS/MS	4 MCD 4 FSGS	CD14, C9, and A1AT ↑ cadherin-like 26, RNase A Family 1, DIS3-like exonuclease 1	Complement activation, inflammation, apoptosis, cells adhesion, and cell death	Choi et al., 2017 [50]
	nano-LC-MS/MS	10 FSGS	↑ Apolipoprotein 1 matrix-remodeling protein 8 (MXRA8)	Lipid oxidation and matrix accumulation	Kalantari et al., 2014 [51]
	nanoLC-MS/MS	11 FSGS	↑ DPEP1, CD59, CD44, insulin-like growth factor-binding protein 7, and roundabout homolog 4	DPEP1 activates TRPC6 in podocytes complement activation, PETs activation, cell–cell interactions, cell adhesion, and maintenance of endothelial barrier organization and function	Nafar et al., 2014 [52]
	MALDI-MS imaging	6 FSGS	A1AT	Marker of podocyte stress, excessive loss, and hypertrophy of podocytes and glomerulosclerosis	Smith et al. 2016 [53]
	CE-MS	110 FSGS 35 MCD	↑ Collagens, A1AT ↓ clusterin, uromodulin polymeric immunoglobulin receptor, Golgi-associated olfactory signaling regulator ↑ collagens, uromodulin, keratin apolipoprotein C-IV ↓ β-2-microglobulin, clusterin, complement C3		Siwy et al., 2017 [54]
	CE-MS	MCD 14 DN 11	retinol-binding protein 4 and SH3 domain-binding glutamic acid-rich-like protein 3	Proteins could distinguish between MCNS and DN	Araumi et al., 2021 [55]
Membranous nephropathy	LC-MS/MS	4	SERPINA7 and CD44	Cell–cell interactions, cell adhesion, and migration	Choi et al., 2017 [50]
	iTRAQ and LC-MS/MS	5	Lysosome membrane protein-2	Immune inflammation	Rood et al., 2015 [56]
	TMT1 and TMT2+nanoLC- MS/MS	63	A1ATafamin	Contribute to accumulation of mesangial matrix lipid metabolism	Pang et al., 2018 [57]
	MALDI-TOF MS	13	↑UMOD ↑A1AT	Tubular dysfunction inflammation and apoptosis; matrix accumulation	Navarro-Muñoz et al., 2012 [58]
	CE-MS	77	↑ A1AT, uromodulin, α-1B-glycoprotein, plasminogen, keratin, apolipoprotein C-IV ↓Fibrinogen α-chain, zinc finger protein ZFPM2, E1A-binding protein Microtubule-associated protein tauAP-3 complex subunit delta-1		Siwy et al., 2017 [54]
	CE-MS	23	The combination of urinary afamin and complement C3 urine/plasma ratio	Could distinguish between MN and DN	Araumi et al., 2021 [55]
IgA nephropathy	iTRAQ-MS	4	Complement C9, Ig kappa chain C region, cytoskeletal keratins type I (10), and type 2 (1, 5)	Complement activation; glomerular filtration barrier damage	Ning et al., 2017 [59]
	2D-LC-MS/MS and iTRAQ	12	ICAM 1, metalloproteinase inhibitor 1, antitrombin III, and adiponectin	Inflammation; urine proteins originated from serum leakage	Guo et al., 2018 [60]
	MALDI-TOF/TOF MS	20	↓ UMOD ↑ A1AT	Accumulation of mesangial matrix	Prikryl et al., 2017 [61]
	CE-MS	209	↓ Collagen I ↑ A1AT	Matrix accumulation and glomerulosclerosis	Rudnicki et al., 2020 [62]
	IEF/LC-MS/MS	30	↑ α-2-macroglobulin, ceruloplasmin, complement C3, complement C4a, haptoglobin, prothrombin, and antithrombin-III	Coagulation, complement activation, and cell interaction in inflammation	Mucha et al., 2014 [63]
	2D-DIGE-MALDI-TOF/TOF	43	Albumin fragments, A1AT, and α-1- β-glycoprotein ↓ laminin G-like 3 (LG3) fragment of endorepellin	Matrix accumulation apoptosis of endothelial cells; extensive fibrosis	Surin et al., 2013 [64]
	nanoLC-MS/MS	13	CD44, glycoprotein 2, vasorin, epidermal growth factor, CMRF35-like molecule 9, protocadherin, utreoglobin, dipeptidyl peptidase IV, NHL repeat-containing protein 3, SLAM family member 5 (CD84)	Activation of apoptosis, immune inflammation, coagulation, and complement	Samavat et al., 2015 [36]
	LC-MS/MS	24	↓ Aminopeptidase N and vasorin precursor levels were higher on average in the urinary exosome samples ↑ A1AT and ceruloplasmin	IgAN markers vs. thin basement membrane nephropathy	Moon et al., 2011 [65]
	CE-MS	179	↑ Small proline-rich protein, leucine-rich repeat-containing protein, A1AT, sodium/potassium-transporting ATPase subunit gamma		Siwy et al., 2017 [54]
Lupus nephritis	SELDI-TOF MS	49 inactive 26 active	Protein ions with m/z of 3340 and 3980	Distinguished active from inactive LN	Mosley et al., 2006 [66]
	SELDI-TOF MS	19 active	Hepcidin, fragments of A1AT, and albumin	Infiltration interstitial leukocytes, cytokines production, and matrix accumulation	Zhang et al., 2008 [67]
	CE-MS	92	Collagens, uromodulin, protein S100-A9, clusterin, β-2-microglobulin, and α-2-HS-glycoprotein	Matrix accumulation	Siwy et al., 2017 [54]
	2D- DIGE-MALDI-TOF MS/MS	88	Haptoglobin, α-1 anti-chymotrypsin, and retinol-binding protein	Effect on inflammation loss of proximal renal tubule function	Aggarwal et al., 2017 [68]
	iTRAQ-MS	61	α1-antichymotrypsin (SERPINA3)	Marker of LN activity	Turnier et al., 2019 [69]
	CE-MS	93	CKD273 validation	It could not identify urinary biomarkers and predict active LN	Tailliar et al., 2021 [70]
Diabetic nephropathy	2D-DIGE-LC-MS/MS	33	↑ α1B-Glycoprotein zinc-α2-glycoprotein, α2-HS-glycoprotein vitamin D–binding protein (VDBP), calgranulin B, A1AT, hemopexin ↓ Transthyretin, apolipoprotein A1, AMBP, and plasma retinol-binding protein	Hyperglycosylated state and matrix accumulation	Rao et al., 2007 [71]
	CE-MS	305	↓ Collagen type I and uromodulin fragments ↑albumin	Increased synthesis of protease inhibitors diminishes excretion of collagen fragments	Rossing et al., 2008 [72]
	CE-MS	126	CKD 273 classifier		Good et al., 2010 [45]
	CE-MS	576	↑ Clusterin, apolipiprotein ↓ hemoglobin, uromodulin, small proline-rich protein 3, leucine-rich repeat-containing protein 25	Accumulation of proteins in the extracellular matrix chronic renal damage	Siwy et al., 2017 [54]
	iTRAQ	50	408 N-linked glycoproteins, A1AT, and ceruloplasmin	Different stage of DN	Jin et al., 2020 [73]
	2D-DIGE-MALDI Q-TOF	268	Transthyretin/prealbumin and Ig kappa C chain region + cystatin C, and ubiquitin + α-1-acid glycoprotein 1, apolipoprotein A1, α-1 microglobulin/bikunin precursor, pigment epithelium-derived factor, zinc α-2 glycoprotein	0–5 years of T2DM duration 5–10 years more than 10 years	Patel and Kalia, 2019 [74]
	iTRAQ	65	↑Haptoglobin and α-1-microglobulin/bikunin precursor		Liao et al., 2018 [75]
	C18 plate–MALDI-TOF	174	↑ β2-microglobulin and clara-cell protein	Proximal tubular dysfunction	Chen et al., 2018 [76]

CE—capillary electrophoresis; DIGE—differential in-gel electrophoresis; ESI—electrospray ionization; IEF—isoelectric focusing; iTRAQ—isobaric tags for relative and absolute quantitation; MALDI—matrix-assisted laser desorption/ionization; SELDI—surface-enhanced matrix assisted laser desorption/ionization; TOF—time-of-flight; TMT—tandem mass tags.

## 2. Chronic Kidney Disease (CKD)

Several urinary proteome studies have considered the CKD group of pathologies without subdivisions. Harald Mischak’s group is the leader in MS studies on the urinary peptidome and proteome. This group described 1580 native urinary peptides, showing that 73% were unique for urine and proving the clinical value of native urinary peptide markers for diagnosing several diseases, including those associated with kidney damage [14,77,78]. 

Rossing K. et al. developed the first panel consisting of 65 urinary proteins, including collagen fragments, serum albumin, α1-antitrypsin (A1AT), and uromodulin, which differentiated diabetic nephropathy in 97% of cases, showing high sensitivity and specificity among 148 type 2 DM and DN patients [72]. The panel was further successfully validated by Alkhalaf A. et al. [79]. 

Good D.M. et al. analyzed urine samples from 476 patients with CKD (mostly diabetic nephropathy) and 379 controls and developed a classifier based on 273 urinary peptides (CKD273) in the form of a composite CKD biomarker [45]. The panel contained fragments of collagen type I and III α-chains (181 peptides), reflecting the extracellular matrix turnover and reduced protease activity in situ. CKD patients also demonstrated increased urinary excretion of plasma proteins and their fragments (e.g., A1AT, serum albumin, α-hemoglobin chain, and α-fibrinogen chain), kidney-specific proteins (uromodulin, gamma-chain Na^+^/K^+^-ATPase, and membrane-associated progesterone receptor component 1), and proteins excreted by the tubules, which may reflect chronic damage to the glomerular filtration barrier, increased glomerulosclerosis, and interstitial fibrosis. In a blinded study, the CKD273 classifier made it possible to differentiate patients with CKD of various etiologies with 85.5% sensitivity and 100% specificity and predict the mortality in type 2 DM with microalbuminuria [45,80,81]. These results were further validated by Schanstra J.P. et al. [18] and Pontillo C. et al. [46], who confirmed this classifier’s value as a predictor of renal function deterioration and demonstrated a decrease in the glomerular filtration rate of <60 mL/min over 5 years of monitoring. Zürbig P. et al. showed that the CKD273 classifier could predict the development of diabetic nephropathy 1.5 years before the onset of microalbuminuria. Argiles A. et al. used the CKD273 classifier on 53 patients with CKD and differentiated patients according to their degrees of impairment of renal function and the risk of end-stage CKD or death [17]. 

Catanese L. et al. developed the FPP_29BH classifier, which contains 29 specific fibrosis biomarkers for patients with various immune and non-immune kidney diseases. The patients with renal fibrosis showed an increase in urinary proteases (cathepsin D, matrix metalloproteinase 2, collagenase 3, and matrix metalloproteinase-14), α-2-HS-glycoprotein, or fetuin-A, as well as 19 different collagen peptide fragments of eight different collagen chains with differential intensities between patients with high and low degrees of fibrosis [47]. 

Since CKD is an umbrella term for several conditions that affect the kidneys, many of the aforementioned markers are not disease-specific. A study of 1180 urine samples by Siwy J. et al. showed that many markers remain the same in different nephropathies and reflect common pathological processes [54]. However, this large-scale study identified a number of specific markers. Three fragments of clusterin were shown to be increased in diabetic nephropathy, β-2-microglobulin was decreased in minimal-change disease, and a S100-A9 protein fragment distinguished lupus nephritis [54]. Other specific proteomic and peptidomic changes in various CKD types, including minimal-change disease (MCD), focal segmental glomerulosclerosis (FSGS), membranous nephropathy (MN), IgA nephropathy (IgAN), diabetic nephropathies (DN), and lupus nephritis (LN), are reviewed below.

## 3. Minimal Change Disease and Focal Segmental Glomerulosclerosis

Minimal-change disease (MCD) and primary focal glomerulosclerosis (FSGS) are diseases with primary podocyte injury (primary podocytopathies), which is manifested as high proteinuria and nephrotic syndrome [82,83]. However, morphological studies of a kidney biopsy in the early stages of FSGS can miss segmental sclerosis in individual glomeruli and may misclassify the disease as MCD [84]. Primary FSGS pathogenesis is associated with circulating permeability factors (such as soluble urokinase-type plasminogen activator receptor (SuPAR), cardiotrophin-like protein-1, and anti-CD40 antibody CD-80 expression), which leads to the development of nephrotic syndrome [85,86,87,88,89,90,91]. In general, compared to FSGS, MCD has a more favorable prognosis regarding the progression of renal dysfunction; FSGS is more likely to develop therapy resistance and result in rapid renal dysfunction and is also more likely to need an aggressive and persistent therapeutic strategy [83,92,93]. In addition, the presence of secondary FSGS complicates diagnosis and disease treatment. Due to its non-immune nature, this form of the disease does not require immunosuppressive therapy [1].

Several studies have aimed at identifying proteomic differences between these two nephropathies. In particular, it was shown that the calretinin and UBA52 levels were higher in FSGS [48,49], while the 39S ribosomal protein L17 was higher in MCD [48] (Table 2). Significantly higher levels of cathepsin B, cathepsin C, and annexin A3 were shown in cases of the collapsing variant of FSGS (characterized by glomerular collapse and a rapid loss of renal function) than in MCD, MN, and other FSGS variants [94]. Several potential markers specific only for FSGS include increased levels of cadherin-like 26, RNase A family 1, DIS3-like exonuclease 1 [50], matrix-remodeling protein 8 [51], CD59, insulin-like growth factor-binding protein 7, and roundabout homolog 4 [52], as well as a decrease in the polymeric immunoglobulin receptor and Golgi-associated olfactory signaling regulator [54] or the complete absence of dipeptidase 1 (DPEP1) [52]. Increased CD14 levels were found to be specific only for MCD [50] and were not identified in any other nephropathy (Table 2). At the same time, increases in transferrin and histatin-3 may distinguish both FSGS and MCD [48] from other types of kidney disease. 

Among the revealed potential markers, the overrepresentation of ribonuclease 2 and underrepresentation of haptoglobin may suggest the worst FSGS prognosis, whereas apolipoprotein A1 and matrix-remodeling protein 8 (MXRA8) showed significant changes between steroid-sensitive and steroid-resistant forms of FSGS [51]. 

In general, the presence of most of the aforementioned proteins in the urine and increases in their levels may reflect massive cell death and the release of intracellular contents during the podocytes’ separation from the glomerular membrane. These results may also suggest special roles for immunity, inflammation, and apoptosis in the development of FSGS. Cell proliferation, differentiation, and death may be involved in MCD development [95]. Dynamic studies performed using a focal segmental glomerulosclerosis rat model (adriamycin (ADR)-induced nephropathy) revealed a gradual increase in afamin and ceruloplasmin, as well as a gradual decrease in cadherin-2 and aggrecan core protein in FSGS, and suggested that decreased levels of fetuin-B, α-1-microglobulin, and α-2-HS-glycoprotein may be promising markers for the early detection of FSGS [96]. Other promising markers include CD44, MXRA8, cathepsins, and apolipoprotein A1. CD44 reflects the activation of parietal epithelial cells, which triggers glomerulosclerosis. MXRA8 cathepsins are involved in fibrosis accumulation and disease progression. Apolipoprotein A1 reflects oxidative stress, is associated with hyperlipidemia, and represents one of the pathogenetic factors in the development of FSGS.

## 4. Membranous Nephropathy

Membranous nephropathy (MN) is a leading cause of nephrotic syndrome (NS) in adults. This disease has an autoimmune nature, which was confirmed by the presence of autoantibodies to podocyte antigens, including antibodies to phospholipase A2 receptors (aPLA2R) and thrombospondin 1 domain-containing 7A (THSD7A) [97,98]. The secondary causes of MN include drug use, infections, autoimmune diseases, and cancer [99]. The primary mechanism of MN is podocyte autoimmune damage by phospholipase A2 receptor antibodies, leading to massive proteinuria. The diagnosis and treatment of this disease are currently based on the determination of the aPLA2R antibody titer. The search for additional markers seems promising in the aPLA2R-negative type of idiopathic MN.

MN patient studies provide comparative cross-sectional analyses of the proteome in MN compared to that in other nephrotic types of nephritis and healthy controls. The panel of specific urinary protein markers distinguishing MN from other nephropathies includes decreased levels of zinc finger protein ZFPM2, E1A-binding protein, and microtubule-associated protein tauAP-3 complex subunit delta-1 [54], as well as increased levels of thyroxine-binding globulin (SERPINA7) [50], lysosome membrane protein-2 (LIMP-2) [56], plasminogen [54], LDB3, PDLI5 [100], and afamin [55,57]. A comparison of samples from patients with APLA2R-positive MN and APLA2R-negative MN, as well as healthy individuals, revealed significantly higher levels of A1AT and afamine in the positive-MN group [101]. A combination of urinary retinol-binding protein 4 and SH3 domain-binding glutamic acid-rich-like protein 3 can differentiate MCD from DN. Similarly, a combination of urinary afamin and complement C3 urine/plasma ratio can differentiate MN from DN [55].

In general, markers found in MN play a role in the classical pathway of complement activation and immune responses, cell adhesion, receptor-mediated endocytosis, platelet degranulation, and the coagulation cascade [57]. LIMP-2 plays a pivotal role in inflammatory immune-response regulation in the kidney tissue [56] and reflects tissue infiltration by immune cells. LIMP-2 may also help to determine disease activity. The LDB3 and PDLI5 proteins play a role in the modification of the podocyte cytoskeleton, which can lead to proteinuria. Afamin, whose elevation is associated with idiopathic MN, is the most promising specific MN marker, as its significance was confirmed in several studies (Table 2).

## 5. IgA Nephropathy

IgA nephropathy (IgAN) is the most common form of chronic glomerular disease in adults. In Europe, the frequency of IgAN ranges from 19 to 51% of the renal biopsies performed for glomerular diseases [102,103,104]. Patients with IgAN often have increased levels of IgA1 with galactose-deficient O-glycans in the hinge region. The blood levels of an aberrantly glycosylated IgA1 are higher in IgAN than in healthy controls or patients with other kidney diseases. The production of galactose-deficient IgA1 antibodies, immune-complex formation, and the accumulation of these complexes in the mesangium were shown to initiate renal injury [105]. Moreover, the activation of alternative complement pathways potentiated tissue injury [106]. Transferrin receptor (CD71) on human mesangial cells can bind immune complexes containing galactose-deficient IgA [107].

About 40 urinary protein markers differentiating IgAN have been described, >20 of which are specific only for IgAN (Table 2). The levels of complement C9, Ig kappa chain C region, and three cytoskeleton keratins (type I(10) and type II (1 and 5)) changed synchronously in the glomeruli (biopsy sample) of IgAN patients compared to the intact renal-tissue areas of patients with tumors [59]. Altered levels of 30 urine proteins and four potential markers (intercellular adhesion molecule 1 (ICAM1), metalloproteinase inhibitor 1, antithrombin III, and adiponectin) were revealed in IgAN with low proteinuria (<1 g/L) and stable renal function (glomerular filtration rate: 57.3 (23–106) mL/min). A larger multicenter study suggested that a decreased number of collagen fragments in the urine (specifically type I collagen) might be most informative in progressive IgAN, due to decreased collagen degradation and collagenase inhibition in kidney fibrosis [62]. 

Other potential IgAN-specific markers include increased levels of adiponectin [60], α2-macroglobulin, complement C4a, prothrombin [63], antithrombin III [60,63], α-1B-glycoprotein [64], glycoprotein 2, epidermal growth factor, CMRF35-like molecule, protocadherin, utreoglobin, dipeptidyl peptidase IV, NHL repeat-containing protein 3, and CD84 [36] and decreased levels of fibulin-5, YIP1 family member 3, prasoposin [108], aminopeptidase N [65], and the LG3 fragment of endorepellin [64]. The last was the only decreased protein in heavier IgAN with a lower glomerular filtration rate [64]. At the same time, high LG3 levels could inhibit angiogenesis and be responsible for renal function loss in some other IgAN patients [64]. Although data on changes in the level of vasorin are inconsistent [36,65], it can also be considered a specific IgAN marker. Antithrombin III is especially noteworthy as the only specific IsAN marker confirmed in two independent studies [60,63].

## 6. Diabetic Nephropathy

Diabetic nephropathy (DN) affects about 30–40% of diabetes mellitus (DM) patients and is the leading cause of CKD and end-stage renal disease (ESRD) all over the world, especially in high- and middle-income countries. DN leads to glomerular mesangial expansion; the thickening of the basement membrane; and, characteristically, the progression of nodular glomerulosclerosis due to glomerular hyperfiltration [109].

The array of potential specific DN markers in the urine includes >10 proteins (Table 2), with increased levels of vitamin D–binding protein, calgranulin B, hemopexin [71], zinc-α2-glycoprotein [71,74], 408 N-linked glycoproteins [73], cystatin C, ubiquitin, α-1-acid glycoprotein 1, pigment epithelium-derived factor [74], Clara cell protein CC16 [76], and fibronectin [110], as well as decreased levels of transthyretin [71,74] and differently changing levels of the α-1 microglobulin/bicunin precursor (AMBP) [71,74,75].

Significant increases in the urinary levels of α1B-glycoprotein (7-fold), zinc-containing α2-glycoprotein (5.9-fold), α2-HS-glycoprotein (4.7-fold), vitamin D-binding protein (4.8-fold), calgranulin B (3.9-fold), A1AT (2.9-fold), and hemopexin (2.4-fold) reliably distinguished DN with macroalbuminuria from DM without albuminuria [71]. Conversely, a significant decrease in transthyretin (4.3-fold), apolipoprotein A1 (3.2-fold), AMBP (1.6-fold), and retinol-binding plasma protein (1.52-fold) was observed in DN with macroalbuminuria [71]. A model study with selected proteins suggested the significance of cathepsin A, mucin 1, the GM2 ganglioside activator, SPARC-like protein 1, and lysosomal acid phosphatase in the poor prognosis of the early development of DN, as well as in kidney fibrosis [111]. A combination of 408 N-linked glycoprotein, A1AT, and ceruloplasmin was shown to be able to distinguish microalbuminuria and normalbuminuria in DN patients [73]. Urinary haptoglobin and AMBP can differentiate between diabetic patients with and without DN [75]. The increased excretion of 15.8 kDa Clara cell protein CC16 was found to be associated with proximal tubule dysfunction in DM patients with micro- or macroalbuminuria compared to DM patients without albuminuria and healthy controls [76]. The levels of osteopontin and fibronectin were also higher in DN compared to those in DM, and increases in urinary neprilysin and VCAM-1 were observed after losartan treatment in DN [110].

A longitudinal study of type 2 DM revealed an increase in urine transthyretin/prealbumin and the Ig kappa C chain region within 0–5 years of the onset of DM; the appearance of cystatin C and ubiquitin after 5–10 years; and the detection of α-1-acid glycoprotein 1, apolipoprotein A1, AMBP, pigment epithelium-derived factor, and zinc α-2-glycoprotein after 10–20 years [74]. The nonenzymatic glycation of these proteins and their peptides interferes with normal tubular reabsorption and may lead to damage to the proximal tubules and the direct excretion of the proteins into urine. 

Overall, the aforementioned DN markers may reflect the processes of tubular atrophy and tubulointerstitial fibrosis, many of which are important for DN prognosis. Zinc-α2-glycoprotein, transthyretin, and AMBP should be especially noted, as their prognostic significance was confirmed in at least two independent studies [71,74,75].

## 7. Lupus Nephritis

Lupus nephritis (LN) is one of the most common and severe complications of systemic lupus erythematosus and usually appears at least 3-5 years after onset of the disease. The mechanisms of renal glomerulus damage can be found in the deposition of immune complexes or autoantibodies with subsequent complement activation [112]. LN leads to severe kidney damage that advances to end-stage renal disease if not treated adequately. The most important goal for LN treatment is to dynamically assess the degree of renal damage activity since the available activity markers (daily proteinuria, erythrocyturia, complement, and antinuclear antibodies) are not informative. LN patients currently need to undergo several kidney biopsies to monitor LN activity during immunosuppressive therapy to determine where LN treatment should be continued or canceled. In this case, there is a need for highly sensitive and specific LN markers able to predict disease exacerbation or indicate insufficient effectiveness of the therapy.

Only a few potential urinary protein markers specific for LN can be noted (Table 2). A pair of peptides, “3340” and “3980” (*m*/*z*), have made it possible to differentiate an acute LN condition from LN remission with 92% sensitivity and 92% specificity prior to any changes in clinical parameters (the urinary protein/creatinine ratio, antibodies to DNA, hematuria, serum creatinine, etc.). Moreover, these peptides were able to predict early relapse and remission [66].

Particular fragments of hepcidin, together with fragments of A1AT and albumin, were found to be more significant than systemic lupus erythematosus renal flare cycle LN in a dynamic study on the urinary proteome [67]. The altered expression of hepcidin 20 might be a marker of renal flare, whereas an increase in hepcidin 25 upon treatment could be used to estimate the effectiveness of therapy [67].

The classifier based on 172 peptides reliably differentiated 92 LN cases from the general CKD group (1180 patients) and identified the protein S100-A9 as another specific LN marker, whose increased level was found to be essential for LN differentiation in combination with increased levels of collagen peptides and uromodulin, as well as decreased levels of clusterin, β-2-microglobulin, and α-2-HS-glycoprotein [54].

α-1-Antichymotrypsin (SERPINA3) is another potential specific LN marker in urine and the only LN marker whose significance was confirmed in two independent studies [68,69]. Together with haptoglobin and retinol-binding protein, SEPINA3 was significantly increased in active LN compared to inactive LN [68]. Moreover, SERPINA3 demonstrated a moderately positive correlation with LN histological activity, which was confirmed via immunohistochemistry [69].

In general, the described LN markers make it possible to assess the activity of the disease and the accumulation of fibrosis in the kidneys, which are very important in clinical practice when managing patients. The increased levels of some proteins may suggest tubular dysfunction during the acute form of the disease [68].

## 8. Non-Specific Urinary Protein Markers

Uromodulin, collagens, A1AT, and their fragments are the main non-specific urine protein markers that were identified in all the aforementioned nephropathies (Table 2), as well as in many other disorders associated with renal disfunction or proteinuria [17,18,19,20,21,22,23,24,25,26,27,28,29,30,31,32,33,34,35,36,37,38,39]. Uromodulin is a kidney-specific glycosylphosphatidylinositol (GPI)-anchored glycoprotein exclusively produced by the epithelial cells lining the thick ascending limb of the loop of Henle and is a normal component of the urine. Collagen peptides are also normally present in urine and reflect the turnover of the extracellular matrix in kidney tissues. Nevertheless, both usual urine components may indicate pathological changes. Uromodulin may also be a potential biomarker relevant to tubular function and CKD [113]. The level of collagen fragments strongly correlates with the initiation of DN [13,17,19,45,72]; quantitative changes in these fragments in urine were noted 3-5 years prior to the development of macroalbuminuria [19]. Overall, the qualitative composition of the collagen fragments can vary in different nephropathies [45,47,54,72]. 

Unlike uromodulin and collagen peptides, the appearance of A1AT in urine is always associated with some type of pathology and may reflect podocyte stress [53]. Notably, an increase in urinary A1AT was observed in all the nephropathies reviewed in the present study (Table 2). 

In general, the assessment of nonspecific markers in combination with specific markers significantly improved the differentiation of nephropathies. In particular, the levels of six UMOD and A1AT peptides differentiated between proliferative and nonproliferative (including MCD, MN, FSGS, and IgAN) forms of glomerular kidney diseases [58]. Moreover, uromodulin overexpression was shown to predispose one to CKDs such as hypertensive nephropathy and DN [114]. The detection of collagen fragments together with the LG3 fragment of endorepellin is crucial for diagnosing IgAN, as collagen may indicate a more severe disease course with impaired angiogenesis and the rapid development of kidney fibrosis [64]. Estimating the levels of A1AT, uromodulin, transferrin, serum albumin, and α-1-β-glycoprotein is also important in IgAN, as such levels reflect common pathological processes, including enhanced apoptosis, inflammation, coagulation, and complement activation [45,54,61,62,64,65,72].

## 9. Conclusions

The research results indicate the great potential of proteomic analysis for the non-invasive diagnosis of kidney diseases, clarification of the leading pathogenetic mechanisms of disease progression, and determination of targets of action for inhibiting disease progression. Unlike kidney biopsy, urine proteomic analysis is safe and reliable, and can be repeated multiple times for the monitoring of disease. The proteomic urinary profile provides valuable information about the leading pathological processes occurring in renal tissues at the time of examination.

The main feature of proteomic analysis is that many of the markers detected in urine are observed as the result of protein penetration from the blood (albumin, retinol-binding protein, etc.) or as reflections of common pathological processes such as extracellular matrix accumulation (collagens and A1AT), the deposition of immunoglobulin complexes, complement activation, apoptosis, lipid oxidation, and tubular dysfunction (β-2-microglobulin, uromodulin, etc.) with high proteinuria. In this case, it is crucial to assess quantitative changes in these indicators to accurately reflect the process activity and damage severity.

One of the most important goals of urine proteomic analysis in patients with CKD is determining disease-specific biomarkers or their combinations. Proteins extracted for the first time warrant the most attention, as they may reflect the most important pathogenetic stages in disease development. For example, CD44, a marker of activated parietal epithelial cells, may reflect the processes of glomerulosclerosis in MN [50] or IgAN [38] but, at the same time, may also be an essential feature for differentiating FSGS from MCD [52]. DPEP1, primarily identified in FSGS, is thought to reflect TRPC6 activation in podocytes [52]; ubiquitin-60S ribosomal protein L40 (UBA52), which is a marker of cellular stress; or components of the podocyte cytoskeleton that are damaged by antibodies [49,115]. Apolipoproteins, which can play a potential role in FSGS pathogenesis as “permeability factors” [116], as well as proteins whose roles are not yet completely understood, such as lysosome membrane protein-2 and afamin in MN [56,57] and the laminin G-like 3 (LG3) fragment of endorepellin in IgAN [64], may reflect pathological processes and could become targets for new approaches to immunosuppressive or nephroprotective therapy. In addition, positive dynamic changes in the proteomic profile after the designated therapy may help to confirm whether the prescribed medications were chosen correctly and are helping to achieve the desired outcomes. However, despite the validation of the CKD 273 classifier in several studies, there is a need to further develop new panels with increased specificity for specific nephropathies. This seems to be the most important goal for further proteomics research.

**Table 2:** Potential urine proteome markers in different nephropathies.

Potential Urine Protein Marker	Nephropaty						
	CKD	FSGS	MCD	MN	IgAN	LN	DN
α-1-antitrypsin (A1AT)	↑ [45]	↑ [48,53,54]	↑ [48,50]	↑ [54,57,58,82]	↑ [54,61,62,64,65]	↑ [67]	↑ [71,73]
Serum albumin	↑ [45]				↑ [64]	↑ [67]	↑ [72,74]
Hemoglobin	↑ [45]						↓ [54]
Fibrinogen α chain	↑ [45]			↓ [54]			
Uromodulin	↑ [45]	↓ [54]	↑ [54]	↑ [54,58]	↓ [61]	↑ [54]	↓ [54,72]
Na^+^/K^+^-ATPase γ chain	↑ [45]				↑ [54]		
Membrane-associated progesterone receptor component 1	↑ [45]						
Collagens	↓ [45] ↑ [47]	↑ [54]	↑ [54]		↓ [45,62,72]	↑ [54]	↓ [72]
Cathepsin D	↑ [47]						
MMP-2	↑ [47]						
MMP-14	↑ [47]						
Collagenase 3	↑ [47]						
α-2-HS-glycoprotein	↑ [47]					↑ [54]	↑ [71]
Fetuin-A	↑ [47]						
Cathepsin B		↑ [94]					
Cathepsin C		↑ [94]					
Annexin A3		↑ [94]					
Transferrin		↑ [48]	↑ [48]				
Histatin-3		↑ [48]	↑ [48]				
39S ribosomal protein L17 (FSGS/MCD)		↓ [48]	↑ [48]				
Calretinin (FSGS/MCD)		↑ [48]	↓ [48]				
UBA52 (FSGS/MCD)		↑ [49]	↓ [49]				
Cadherin-like 26		↑ [50]					
RNase A Family 1		↑ [50]					
DIS3-like exonuclease 1		↑ [50]					
CD14			↑ [50]				
Complement C9			↑ [50]		↑ [59]		
Apolipoprotein A1		↑ [51]					↓ [71], ↑ [54,74]
Matrix-remodeling protein 8		↑ [51]					
Dipeptidase 1 (DPEP1)		↓ [52]					
CD59		↑ [52]					
CD44		↑ [52]		↑ [50]	↑ [36]		
Insulin-like growth factor-binding protein 7		↑ [52]					
Roundabout homolog 4		↑ [52]					
Clusterin		↓ [54]	↓ [54]			↑ [54]	↑ [54]
Polymeric immunoglobulin receptor		↓ [54]					
Golgi-associated olfactory signaling regulator		↓ [54]					
Apolipoprotein C-IV			↑ [54]	↑ [54]			
β-2-microglobulin			↓ [54]			↑ [54]	↑ [76]
Complement C3			↓ [54]	[55]	↑ [63]		
Retinol-binding protein 4			[55]		[117]		[55]
SH3 domain-binding glutamic acid-rich-like protein 3			[55]				[55]
Thyroxine-binding globulin (SERPINA7)				↑ [50]			
Lysosome membrane protein-2				↑ [105]			
Afamin				↑ [55,57,101]			
α-1B-glycoprotein				↑ [54]	↑ [64]		↑ [71]
Plasminogen				↑ [54]			
Zinc finger protein ZFPM2				↓ [54]			
E1A-binding protein				↓ [54]			
Microtubule-associated protein tauAP-3 complex subunit delta-1				↓ [54]			
LDB3				↑ [100]			
PDLI5				↑ [100]			
Ig kappa chain C region					↑ [59]		↑ [74]
Cytoskeletal keratins type I (10) and type 2 (1, 5)					↑ [59]		
ICAM1					↑ [60]		
Metalloproteinase inhibitor 1					↑ [60]		
Antitrombin III					↑ [60,63]		
Adiponectin					↑ [60]		
α-2-macroglobulin					↑ [63]		
Ceruloplasmin					↑ [63,65]		↑ [73]
Complement C4a					↑ [63]		
Haptoglobin					↑ [63]	↑ [68]	↑ [75]
Prothrombin					↑ [63]		
LG3 fragment of endorepellin					↓ [64]		
Glycoprotein 2					↑ [36]		
Vasorin					↑ [36] ↓ [65]		
Epidermal growth factor					↑ [36]		
CMRF35-like molecule 9					↑ [36]		
Protocadherin					↑ [36]		
Utreoglobin					↑ [36]		
Dipeptidyl peptidase IV					↑ [36]		
NHL repeat-containing protein 3					↑ [36]		
SLAM family member 5 (CD84)					↑ [36]		
Aminopeptidase N					↓ [65]		
Fibulin-5					↓ [108]		
YIP1 family member 3					↓ [108]		
Prasoposin					↓ [108]		
Osteopontin					↓ [108]		↑ [110]
Small proline-rich protein 3					↑ [108]		↓ [54]
Leucine-rich repeat-containing protein 25					↑ [54]		↓ [54]
3340 and 39110 (m/z)						↑ [66]	
Hepcidin						↑ [67]	
Protein S89-A9						↑ [54]	
α-1 anti-chymotrypsin (SERPINA3)						↑ [68,69]	
Retinol binding protein						↑ [68]	↓ [71]
Zinc-α2-glycoprotein							↑ [71,74]
Vitamin D-binding protein							↑ [71]
Calgranulin B							↑ [71]
Hemopexin							↑ [71]
Transthyretin							↓ [71,74]
α-1 microglobulin/bikunin precursor (AMBP)							↓ [71] ↑ [74,75]
408 N-linked glycoproteins							↑ [73]
Cystatin C							↑ [74]
Ubiquitin							↑ [74]
α-1-acid glycoprotein 1							↑ [74]
Pigment epithelium-derived factor							↑ [74]
Clara cell protein CC16							↑ [76]
Fibronectin							↑ [110]

The gray background indicates the most potential markers identified for specific nephropathy in at least 2 studies.

## Data Availability

Not applicable.

## References

[1] Kidney Disease Improving Global Outcomes (KDIGO) (2012). KDIGO Clinical Practice Guideline for Glomerulonephritis.

[2] National Kidney Foundation (2002). K/DOQI clinical practice guidelines for chronic kidney disease: Evaluation, classification, and stratification. Am. J. Kidney Dis..

[3] Sarnak M.J., Levey A.S., Schoolwerth A.C., Coresh J., Culleton B., Hamm L.L., McCullough P.A., Kasiske B.L., Kelepouris E., Klag M.J. (2003). Kidney Disease as a Risk Factor for Development of Cardiovascular Disease. Circulation.

[4] Alani H., Tamimi A., Tamimi N. (2014). Cardiovascular co-morbidity in chronic kidney disease: Current knowledge and future research needs. World J. Nephrol..

[5] Hsu C., Ordoñez J., Chertow G., Fan D., McCulloch C., Go A. (2008). The risk of acute renal failure in patients with chronic kidney disease. Kidney Int..

[6] Tonelli M., Wiebe N., Culleton B., House A., Rabbat C., Fok M., McAlister F., Garg A.X. (2006). Chronic Kidney Disease and Mortality Risk: A Systematic Review. J. Am. Soc. Nephrol..

[7] Hsu C.-Y., Iribarren C., McCulloch C.E., Darbinian J., Go A.S. (2009). Risk Factors for End-Stage Renal Disease: 25-year follow-up. Arch. Intern. Med..

[8] Hill N.R., Fatoba S.T., Oke J.L., Hirst J., O’Callaghan C.A., Lasserson D., Hobbs R. (2016). Global Prevalence of Chronic Kidney Disease—A Systematic Review and Meta-Analysis. PLoS ONE.

[9] Schieppati A., Remuzzi G. (2005). Chronic renal diseases as a public health problem: Epidemiology, social, and economic implications. Kidney Int..

[10] Bommer J. (2002). Prevalence and socio-economic aspects of chronic kidney disease. Nephrol. Dial. Transplant..

[11] Vos T., Allen C., Arora M., Barber R.M., Bhutta Z.A., Brown A., Carter A., Casey D.C., Charlson F.J., Chen A.Z. (2015). Global, regional, and national incidence, prevalence, and years lived with disability for 310 diseases and injuries, 1990–2015: A systematic analysis for the Global Burden of Disease Study 2015. Lancet.

[12] Dhaun N., Bellamy C.O., Cattran D.C., Kluth D.C. (2014). Utility of renal biopsy in the clinical management of renal disease. Kidney Int..

[13] Filip S., Pontillo C., Schanstra J.P., Vlahou A., Mischak H., Klein J. (2014). Urinary proteomics and molecular determinants of chronic kidney disease: Possible link to proteases. Expert Rev. Proteom..

[14] Mischak H., Delles C., Vlahou A., Vanholder R. (2015). Proteomic biomarkers in kidney disease: Issues in development and im-plementation. Nat. Rev. Nephrol..

[15] Decramer S., Gonzalez de Peredo A., Breuil B., Mischak H., Monsarrat B., Bascands J.-L., Schanstra J.P. (2008). Urine in Clinical Proteomics. Mol. Cell. Proteom..

[16] Thomas S., Hao L., Ricke W., Li L. (2016). Biomarker discovery in mass spectrometry-based urinary proteomics. Proteom. Clin. Appl..

[17] Argiles A., Siwy J., Duranton F., Gayrard N., Dakna M., Lundin U., Osaba L., Delles C., Mourad G., Weinberger K.M. (2013). CKD273, a New Proteomics Classifier Assessing CKD and Its Prognosis. PLoS ONE.

[18] Schanstra J.P., Zürbig P., Alkhalaf A., Argiles A., Bakker S.J.L., Beige J., Bilo H.J.G., Chatzikyroku C., Dakna M., Dawson J. (2015). Diagnosis and prediction of CKD progression by assessment of urinary peptides. JASN.

[19] Zürbig P., Jerums G., Hovind P., MacIsaac R.J., Mischak H., Nielsen S.E., Panagiotopoulos S., Persson F., Rossing P. (2012). Urinary Proteomics for Early Diagnosis in Diabetic Nephropathy. Diabetes.

[20] Celis J.E., Gromova I., Moreira J.M., Cabezon T., Gromov P. (2004). Impact of proteomics on bladder cancer research. Pharmacogenomics.

[21] Chen Y.-T., Chen H.-W., Domanski D., Smith D.S., Liang K.-H., Wu C.-C., Chen C.-L., Chung T., Chen M.-C., Chang Y.-S. (2012). Multiplexed quantification of 63 proteins in human urine by multiple reaction monitoring-based mass spectrometry for discovery of potential bladder cancer biomarkers. J. Proteom..

[22] Shi T., Gao Y., Quek S.I., Fillmore T.L., Nicora C.D., Su D., Zhao R., Kagan J.L., Srivastava S., Rodland K.D. (2014). A Highly Sensitive Targeted Mass Spectrometric As-say for Quantification of AGR2 Protein in Human Urine and Serum. J. Proteom. Res..

[23] Ye B., Skates S., Mok S.C., Horick N.K., Rosenberg H.F., Vitonis A., Edwards D., Sluss P., Han W.K., Berkowitz R.S. (2006). Proteomic-Based Discovery and Characterization of Glycosylated Eosinophil-Derived Neurotoxin and COOH-Terminal Osteopontin Fragments for Ovarian Cancer in Urine. Clin. Cancer Res..

[24] Mischak H., Kaiser T., Walden M., Hillmann M., Wittke S., Herrmann A., Knueppel S., Haller H., Fliser D. (2004). Proteomic analysis for the assessment of diabetic renal damage in humans. Clin. Sci..

[25] Buhimschi I.A., Zhao G., Funai E.F., Harris N., Sasson I.E., Bernstein I.M., Saade G.R., Buhimschi C.S. (2008). Proteomic profiling of urine identifies specific fragments of SERPINA1 and albumin as biomarkers of preeclampsia. Am. J. Obstet. Gynecol..

[26] Carty D.M., Siwy J., Brennand J.E., Zürbig P., Mullen W., Franke J., McCulloch J.W., North R.A., Chappell L.C., Mischak H. (2011). Urinary Proteomics for Prediction of Preeclampsia. Hypertension.

[27] Kononikhin A.S., Zakharova N.V., Sergeeva V.A., Indeykina M.I., Starodubtseva N.L., Bugrova A.E., Muminova K.T., Khodzhaeva Z.S., Popov I.A., Shao W. (2020). Differential Diagnosis of Preeclampsia Based on Urine Peptidome Features Revealed by High Resolution Mass Spectrometry. Diagnostics.

[28] Ward D.G., Nyangoma S., Joy H., Hamilton E., Wei W., Tselepis C., Steven N., Wakelam M.J., Johnson P.J., Ismail T. (2008). Proteomic profiling of urine for the detection of colon cancer. Proteom. Sci..

[29] Tantipaiboonwong P., Sinchaikul S., Sriyam S., Phutrakul S., Chen S.-T. (2005). Different techniques for urinary protein analysis of normal and lung cancer patients. Proteomics.

[30] Metzger J., Negm A.A., Plentz R.R., Weismüller T.J., Wedemeyer J., Karlsen T.H., Dakna M., Mullen W., Mischak H., Manns M.P. (2012). Urine proteomic analysis differentiates cholangiocarcinoma from primary sclerosing cholangitis and other benign biliary disorders. Gut.

[31] Zimmerli L.U., Schiffer E., Zürbig P., Good D.M., Kellmann M., Mouls L., Pitt A.R., Coon J.J., Schmeider R.D., Peter K.H. (2008). Urinary Proteomic Biomarkers in Coronary Artery Disease. Mol. Cell. Proteom..

[32] Kaiser T., Kamal H., Rank A., Kolb H.-J., Holler E., Ganser A., Hertenstein B., Mischak B., Weisseinger M.E. (2004). Proteomics applied to the clinical follow-up of pa-tients after allogeneic hematopoietic stem cell transplantation. Blood.

[33] Taneja S., Sen S., Gupta V.K., Aggarwal R., Jameel S. (2009). Plasma and urine biomarkers in acute viral hepatitis E. Proteome Sci..

[34] Kalantari S., Jafari A., Moradpoor R., Ghasemi E., Khalkhal E. (2015). Human urine proteomics: Analytical techniques and clini-cal applications in renal diseases. Int. J. Proteom..

[35] Fang X., Wu H., Lu M., Cao Y., Wang R., Wang M., Gao C., Xia Z. (2020). Urinary proteomics of Henoch-Schönlein purpura nephri-tis in children using liquid chromatography-tandem mass spectrometry. Clin. Proteom..

[36] Samavat S., Kalantari S., Nafar M., Rutishauser D., Rezaei-Tavirani M., Parvin M., Zubarev R.A. (2015). Diagnostic Urinary Pro-teome Profile for Immunoglobulin A Nephropathy. Iran. J. Kid. Dis..

[37] Cunningham R., Ma D., Li L. (2012). Mass spectrometry-based proteomics and peptidomics for systems biology and biomarker discovery. Front. Biol..

[38] Di Meo A., Pasic M.D., Yousef G.M. (2016). Proteomics and peptidomics: Moving toward precision medicine in urological malignancies. Oncotarget.

[39] Feist P., Hummon A.B. (2015). Proteomic Challenges: Sample Preparation Techniques for Microgram-Quantity Protein Analysis from Biological Samples. Int. J. Mol. Sci..

[40] Khan A., Packer N. (2006). Simple Urinary Sample Preparation for Proteomic Analysis. J. Proteom. Res..

[41] Tanaka T., Biancotto A., Moaddel R., Moore A.Z., Gonzalez-Freire M., Aon M.A., Candia J., Zhang P., Cheung F., Fantoni G. (2018). Plasma proteomic signature of age in healthy humans. Aging Cell.

[42] Shao C., Zhao M., Chen X., Sun H., Yang Y., Xiao X., Guo Z., Liu X., Lv Y., Chen X. (2019). Comprehensive Analysis of Individual Variation in the Urinary Proteome Revealed Significant Gender Differences. Mol. Cell. Proteom..

[43] Nkuipou-Kenfack E., Bhat A., Klein J., Jankowski V., Mullen W., Vlahou A., Dakna M., Koeck T., Schanstra J.P., Zürbig P. (2015). Identification of ageing-associated naturally occurring peptides in human urine. Oncotarget.

[44] Mischak H., Ioannidis J.P., Argiles A., Attwood T., Bongcam-Rudloff E., Brönstrup M., Charonis A., Chrousos G.P., Delles C., Dominiczak A. (2012). Implementation of proteomic biomarkers: Making it work. Eur. J. Clin. Investig..

[45] Good D.M., Zürbig P., Argilés A., Bauer H.W., Behrens G., Coon J.J., Dakna M., Decramer S., Delles C., Dominiczak A.F. (2010). Naturally occurring human urinary peptides for use in diagnosis of chronic kidney disease. Mol. Cell. Proteom..

[46] Pontillo C., Zhang Z.-Y., Schanstra J.P., Jacobs L., Zürbig P., Thijs L., Ramírez-Torres A., Heerspink H.J., Lindhardt M., Klein R. (2017). Prediction of Chronic Kidney Disease Stage 3 by CKD273, a Urinary Proteomic Biomarker. Kidney Int. Rep..

[47] Catanese L., Siwy J., Mavrogeorgis E., Amann K., Mischak H., Beige J., Rupprecht H. (2021). A Novel Urinary Proteomics Classifier for Non-Invasive Evaluation of Interstitial Fibrosis and Tubular Atrophy in Chronic Kidney Disease. Proteomes.

[48] Pérez V., López D., Boixadera E., Ibernón M., Espinal A., Bonet J., Romero R. (2017). Comparative differential proteomic analysis of minimal change disease and focal segmental glomerulosclerosis. BMC Nephrol..

[49] Wang Y., Zheng C., Wang X., Zuo K., Liu Z. (2017). Proteomic profile-based screening of potential protein biomarkers in the urine of patients with nephrotic syndrome. Mol. Med. Rep..

[50] Choi Y.W., Kim Y.G., Song M.-Y., Moon J.-Y., Jeong K.-H., Lee T.-W., Ihm C.-G., Park K.-S., Lee S.-H. (2017). Potential urine proteomics biomarkers for primary nephrotic syndrome. Clin. Proteom..

[51] Kalantari S., Nafar M., Samavat S., Rezaei-Tavirani M., Rutishauser D., Zubarev R. (2014). Urinary Prognostic Biomarkers in Patients With Focal Segmental Glomerulosclerosis. Nephro-Urol. Mon..

[52] Nafar M., Kalantari S., Samavat S., Rezaei-Tavirani M., Rutishuser D., Zubarev R.A. (2014). The novel diagnostic bi-omarkers for focal segmental Glomerulosclerosis. Int. J. Nephrol..

[53] Smith A., L’Imperio V., De Sio G., Ferrario F., Scalia S., Dell’Antonio G., Pierrutzzi F., Pontillo C., Filip S., Markoska A. (2016). α-1-Antitrypsin detected by MALDI imaging in the study of glomerulonephritis: Its relevance in chronic kidney disease progression. Proteomics.

[54] Siwy J., Zürbig P., Argiles A., Beige J., Haubitz M., Jankowski J., Julian B.A., Linde P.B., Marx D., Mishkac H. (2017). Noninvasive diagnosis of chronic kidney diseases using urinary proteome analysis. Nephrol. Dial. Transplant..

[55] Araumi A., Osaki T., Ichikawa K., Kudo K., Suzuki N., Watanabe S., Watanabe M., Konta T. (2021). Urinary and plasma proteomics to discover biomarkers for diagnosing between diabetic nephropathy and minimal change nephrotic syndrome or mem-branous nephropathy. Biochem. Biophys. Rep..

[56] Rood I.M., Merchant M.L., Wilkey D.W., Zhang T., Zabrouskov V., van der Vlag J., Dijikman H.B., Wilemens B.K., Wetzles J.F., Klein J.B. (2015). Increased expression of lysosome membrane protein 2 in glomeruli of patients with idiopathic membranous nephropathy. Proteomics.

[57] Pang L., Li Q., Li Y., Liu Y., Duan N., Li H. (2018). Urine proteomics of primary membranous nephropathy using nanoscale liquid chromatography tandem mass spectrometry analysis. Clin. Proteom..

[58] Navarro-Muñoz M., Ibernon M., Bonet J., Pérez V., Pastor M.C., Bayés B., Casado-Vela J., Navarro M., Ara J., Espinal A. (2012). Uromodulin and α1-Antitrypsin Urinary Peptide Analysis to Differentiate Glomerular Kidney Diseases. Kidney Blood Press. Res..

[59] Ning X., Yin Z., Li Z., Xu J., Wang L., Shen W., Lu Y., Cai G., Zhang X., Chen X. (2017). Comparative proteomic analysis of urine and laser microdissected glomeruli in IgA nephropathy. Clin. Exp. Pharmacol. Physiol..

[60] Guo Z., Wang Z., Lu C., Yang S., Sun H., Reziw, Guo Y., Sun W., Yue H. (2018). Analysis of the differential urinary protein profile in IgA nephropathy patients of Uygur ethnicity. BMC Nephrol..

[61] Prikryl P., Vojtova I., Maixnerova D., Vokurka M., Neprasova M., Zima T., Tesar V. (2017). Proteomic Approach for Identification of IgA Nephropathy-Related Biomarkers in Urine. Physiol. Res..

[62] Rudnicki M., Siwy J., Wendt R., Lipphardt M., Koziolek M.J., Maixnerova D., Peters B., Kerschbaum J., Leierer J., Neprasova M. (2020). Urine proteomics for prediction of disease progression in patients with IgA nephropathy. Nephrol. Dial. Transplant..

[63] Mucha K., Bakun M., Jaźwiec R., Dadlez M., Florczak M., Bajor M., Gala K., Pączek L. (2014). Complement components, proteolysis-related, and cell communication?related proteins detected in urine proteomics are associated with IgA nephropathy. Pol. Arch. Intern. Med..

[64] Surin B., Sachon E., Rougier J.-P., Steverlynck C., Garreau C., Lelongt B. (2013). LG3 fragment of endorepellin is a possible bi-omarker of severity in IgA nephropathy. Proteomics.

[65] Moon P.G., Lee J.E., You S., Kim T.K., Cho J.H., Kim I.S., Kwon T.-H., Kim C.-D., Park S.-H., Hwang D. (2011). Proteomic analysis of urinary exosomes from patients of early IgA nephropathy and thin basement membrane nephropathy. Proteomics.

[66] Mosley K., Tam F.W.K., Edwards R.J., Crozier J., Pusey C.D., Lightstone L. (2006). Urinary proteomic profiles distinguish between active and inactive lupus nephritis. Rheumatology.

[67] Zhang X., Jin M., Wu H., Nadasdy T., Nadasdy G., Harris N., Green-Church K., Nagaraja H., Birmingham D.J., Yu C.-Y. (2008). Biomarkers of lupus nephritis determined by serial urine proteomics. Kidney Int..

[68] Aggarwal A., Gupta R., Negi V.S., Rajasekhar L., Misra R., Singh P., Chaturvedi V., Sinha S. (2017). Urinary haptoglobin, alpha-1 anti-chymotrypsin and retinol binding protein identified by proteomics as potential biomarkers for lupus nephritis. Clin. Exp. Immunol..

[69] Turnier J.L., Brunner H.I., Bennett M., Aleed A., Gulati G., Haffey W.D., Thornton S., Wagner M., Devarajan P., Witte D. (2018). Discovery of SERPINA3 as a candidate urinary biomarker of lupus nephritis activity. Rheumatology.

[70] Tailliar M., Schanstra J., Dierckx T., Breuil B., Hanouna G., Charles N., Bascands J.-L., Dussol B., Vazi A., Chiche L. (2021). Urinary Peptides as Potential Non-Invasive Biomarkers for Lupus Nephritis: Results of the Peptidu-LUP Study. J. Clin. Med..

[71] Rao P.V., Lu X., Standley M., Pattee P., Neelima G., Girisesh G., Dakshinamurthy K.V., Roberts C.T., Nagalla S.S. (2007). Proteomic identification of urinary biomarkers of diabetic nephropathy. Diabetes Care.

[72] Rossing K., Mischak H., Dakna M., Zürbig P., Novak J., Julian B.A., Good D.M., Coon J.J., Tarnow L., Rossing P. (2008). Urinary Proteomics in Diabetes and CKD. J. Am. Soc. Nephrol..

[73] Jin J., Gong J., Zhao L., Li Y., Wang Y., He Q. (2020). iTRAQ-based comparative proteomics analysis reveals specific urinary biomarkers for various kidney diseases. Biomark. Med..

[74] Patel D.N., Kalia K. (2019). Characterization of low molecular weight urinary proteins at varying time intervals in type 2 diabetes mellitus and diabetic nephropathy patients. Diabetol. Metab. Syndr..

[75] Liao W.-L., Chang C.-T., Chen C.-C., Lee W.-J., Lin S.-Y., Liao H.-Y., Wu C.-M., Chang Y.-W., Chen C.-J., Tsai F.-J. (2018). Urinary Proteomics for the Early Diagnosis of Diabetic Nephropathy in Taiwanese Patients. J. Clin. Med..

[76] Chen C.J., Liao W.L., Chang C.T., Liao H.Y., Tsai F.J. (2018). Urine proteome analysis by C18 plate-matrix-assisted laser desorption/ionization time-of-flight mass spectrometry allows non-invasive differential diagnosis and prediction of diabetic nephropathy. PLoS ONE.

[77] He T., Pejchinovski M., Mullen W., Beige J., Mischak H., Jankowski V. (2020). Peptides in Plasma, Urine, and Dialysate: Toward Unravelling Renal Peptide Handling. Proteom. Clin. Appl..

[78] He T., Siwy J., Metzger J., Mullen W., Mischak H., Schanstra J.P., Zurbin P., Jankowski V. (2020). Associations of urinary polymeric immunoglobulin receptor peptides in the context of cardiorenal syndrome. Sci. Rep..

[79] Alkhalaf A., Zürbig P., Bakker S.J.L., Bilo H.J.G., Cerna M., Fischer C., Fuchs S., Jannsen N., Medek C., Miskhac H. (2010). Multicentric validation of proteomic biomarkers in urine specific for diabetic nephropathy. PLoS ONE.

[80] Currie G.E., von Scholten B.J., Mary S., Guerrero J.-L.F., Lindhardt M., Reinhard H., Jacobsen P.K., Mullen W., Parving H.-H., Mischak H. (2018). Urinary proteomics for prediction of mortality in patients with type 2 diabetes and microalbuminuria. Cardiovasc. Diabetol..

[81] Brondani L.D.A., Soares A.A., Recamonde-Mendoza M., Dall’Agnol A., Camargo J.L., Monteiro K.M., Silveiro S.P. (2020). Urinary peptidomics and bioinformatics for the detection of diabetic kidney disease. Sci. Rep..

[82] Praga M., Morales E., Herrero J.C., Campos A.P., Domínguez-Gil B., Alegre R., Vara J., Martínez M.A. (1999). Absence of hypoalbuminemia despite massive proteinuria in focal segmental glomerulosclerosis secondary to hyperfiltration. Am. J. Kidney Dis..

[83] Rydel J.J., Korbet S.M., Borok R.Z., Schwartz M.M. (1995). Focal segmental glomerular sclerosis in adults: Presentation, course, and response to treatment. Am. J. Kidney Dis..

[84] D’Agati V.D., Fogo A.B., Bruijn J.A., Jennette J. (2004). Pathologic classification of focal segmental glomerulosclerosis: A working proposal. Am. J. Kidney Dis..

[85] Rosenberg A.Z., Kopp J.B. (2017). Focal Segmental Glomerulosclerosis. Clin. J. Am. Soc. Nephrol..

[86] Savin V.J., Sharma R., Sharma M., McCarthy E.T., Swan S.K., Ellis E., Lovell H., Warady B., Gunwar S., Chonko A.M. (1996). Circulating Factor Associated with Increased Glomerular Permeability to Albumin in Recurrent Focal Segmental Glomerulosclerosis. N. Engl. J. Med..

[87] Wei C., El Hindi S., Li J., Fornoni A., Goes N., Sageshima J., Karumanchi S.A., Miguel D., Yap H.-K., Saalem M. (2011). Circulating urokinase receptor as a cause of focal segmental glomerulosclerosis. Nat. Med..

[88] Shankland S.J., Pollak M.R. (2011). A suPAR circulating factor causes kidney disease. Nat. Med..

[89] Sharma M., Zhou J., Gauchat J.-F., Sharma R., McCarthy E.T., Srivastava T., Savin V.J. (2015). Janus kinase 2/signal transducer and activator of transcription 3 inhibitors attenuate the effect of cardiotrophin-like cytokine factor 1 and human focal segmental glomerulosclerosis serum on glomerular filtration barrier. Transl. Res..

[90] Delville M., Sigdel T.K., Wei C., Li J., Hsieh S.-C., Fornoni A., Burke G.W., Bruneval P., Naesens M., Jackson A. (2014). A circulating antibody panel for pretransplant prediction of FSGS recurrence after kidney transplantation. Sci. Transl. Med..

[91] Yu C.-C., Fornoni A., Weins A., Hakroush S., Maiguel D., Sageshima J., Chen L., Ciancio G., Faridi M.H., Behr D. (2013). Abatacept in B7-1–Positive Proteinuric Kidney Disease. N. Engl. J. Med..

[92] Korbet S.M., Schwartz M.M., Lewis E.J. (1994). Primary Focal Segmental Glomerulosclerosis: Clinical Course and Response to Therapy. Am. J. Kidney Dis..

[93] Wehrmann M., Bohle A., Held H., Schumm G., Kendziorra H., Pressler H. (1990). Long-term prognosis of focal sclerosing glomerulonephritis. An analysis of 250 cases with particular regard to tubulointerstitial changes. Clin. Nephrol..

[94] Merchant M.L., Barati M.T., Caster D.J., Hata J.L., Hobeika L., Coventry S., Brier M.E., Wilkey D.W., Li M., Rood I.M. (2020). Proteomic Analysis Identifies Distinct Glomerular Extracellular Matrix in Collapsing Focal Segmental Glomerulosclerosis. J. Am. Soc. Nephrol..

[95] Schwaller B. (2014). Calretinin: From a “simple” Ca^2+^ buffer to a multifunctional protein implicated in many biological processes. Front. Neuroanat..

[96] Beeken M., Lindenmeyer M.T., Blattner S.M., Radón V., Oh J., Meyer T.N., Hildebrand D., Schlüter H., Reinicke A.T., Knop J.-H. (2014). Alterations in the Ubiquitin Proteasome System in Persistent but Not Reversible Proteinuric Diseases. J. Am. Soc. Nephrol..

[97] Meyer-Schwesinger C., Meyer T., Münster S., Klug P., Saleem M., Helmchen U., Stahl R. (2009). A new role for the neuronal ubiquitin C-terminal hydrolase-L1 (UCH-L1) in podocyte process formation and podocyte injury in human glomerulopathies. J. Pathol..

[98] Meyer-Schwesinger C., Meyer T.N., Sievert H., Hoxha E., Sachs M., Klupp E.M., Munster S., Balabanov S., Carrier L., Helmchen U. (2011). Ubiquitin C-terminal hydro-lase-l1 activity induces polyubiquitin accumulation in podocytes and increases proteinuria in rat membranous nephropathy. Am. J. Pathol..

[99] Moroni G., Ponticelli C. (2020). Secondary Membranous Nephropathy. A Narrative Review. Front. Med..

[100] Ligabue G., Magistroni R., Cantu’ M., Genovese F., Lupo V., Cavazzini F., Furci L., Cappelli F. (2013). Identification and Characterization of New Proteins in Podocyte Dysfunction of Membranous Nephropathy by Proteomic Analysis of Renal Biopsy. Curr. Pharmacogen. Person. Med..

[101] Dieplinger H., Dieplinger B. (2015). Afamin—A pleiotropic glycoprotein involved in various disease states. Clin. Chim. Acta.

[102] McGrogan A., Franssen C.F., de Vries C.S. (2011). The incidence of primary glomerulonephritis worldwide: A systematic review of the literature. Nephrol. Dial. Transplant..

[103] Zaza G., Bernich P., Lupo A., Triveneto’ Register of Renal Biopsies (TVRRB) (2013). Incidence of primary glomerulonephritis in a large North-Eastern Italian area: A 13-year renal biopsy study. Nephrol. Dial. Transplant..

[104] Maixnerova D., Bauerova L., Skibova J., Rysava R., Reiterova J., Merta M., Honsova E., Tesar V. (2012). The retrospective analysis of 343 Czech patients with IgA nephropathy—One centre experience. Nephrol. Dial. Transplant..

[105] Suzuki H., Kiryluk K., Novak J., Moldoveanu Z., Herr A., Renfrow M.B., Wyatt R., Scolari F., Mestecky J., Gharavi A.G. (2011). The Pathophysiology of IgA Nephropathy. J. Am. Soc. Nephrol..

[106] Zhang W., Lachmann P.J. (1994). Glycosylation of IgA is required for optimal activation of the alternative complement pathway by immune complexes. Immunology.

[107] Moura I.C., Arcos-Fajardo M., Gdoura A., Leroy V., Sadaka C., Mahlaoui N., Yves L., Vrtovnski F., Haddad E., Benhamou M. (2005). Engagement of transferrin receptor by polymeric IgA1: Evidence for a positive feedback loop involving increased receptor expression and mesangial cell proliferation in IgA nephropathy. JASN.

[108] Majd T.M., Kalantari S., Shahraki H.R., Nafar M., Almasi A., Samavat S., Parvin M., Hashemian A. (2018). Application of sparse linear discriminant analysis and elastic net for diagnosis of IgA nephropathy: Statistical and biological viewpoints. Iran. Biomed. J..

[109] Johnson R.J., Feehally J., Floege J. (2019). Comprehensive Clinical Nephrology.

[110] Guillén-Gómez E., de Quixano B.B., Ferrer S., Brotons C., Knepper M.A., Carrascal M., Abian J., Mas J.M., Calero F., Ballarín J.A. (2018). Urinary Proteome Analysis Identified Neprilysin and VCAM as Proteins Involved in Diabetic Nephropathy. J. Diabetes Res..

[111] Ahn H.-S., Kim J.H., Jeong H., Yu J., Yeom J., Song S.H., Kim S.S., Kim I.J., Kim K. (2020). Differential Urinary Proteome Analysis for Predicting Prognosis in Type 2 Diabetes Patients with and without Renal Dysfunction. Int. J. Mol. Sci..

[112] Musa R., Brent L.H., Qurie A. (2021). Lupus Nephritis.

[113] 109Devuyst O., Bochud M. (2015). Uromodulin, kidney function, cardiovascular disease, and mortality. Kidney Int..

[114] Trudu M., Janas S., Lanzani C., Debaix H., Schaeffer C., Ikehata M., Citterio L., Demaretz C., Trevisani F., Ristango G. (2013). Common noncoding UMOD gene variants induce salt-sensitive hypertension and kidney damage by increasing uromodulin expression. Nat. Med..

[115] Jamin A., Berthelot L., Couderc A., Chemouny J.M., Boedec E., Dehoux L., Abbad L., Dossier C., Daugas E., Monteiro R. (2018). Autoantibodies against podocytic UCHL1 are associated with idiopathic nephrotic syndrome relapses and induce proteinuria in mice. J. Autoimmun..

[116] Bruschi M., Catarsi P., Candiano G., Pia M., Rastaldi M.P., Musante L., Scolari F., Artero M., Carraro M., Carrea A. (2003). Apolipoprotein E in idiopathic nephrotic syndrome and focal segmental glomerulosclerosis. Kidney Int..

[117] Marek-Bukowiec K., Konieczny A., Ratajczyk K., Macur K., Czaplewska P., Czyżewska-Buczyńska A., Kowal P., Witkiewicz W. (2020). The value of urinary RBP4 in the diagnosis of FSGS and other renal diseases. Trends Biomed. Res..

